# Adaptive radiation therapy for glioblastoma: clinical efficacy and recurrence patterns

**DOI:** 10.1186/s13014-025-02778-6

**Published:** 2025-12-13

**Authors:** Tomohiko Matsuyama, Shigeo Yamada, Hirohito Otsuka, Takahiro Watakabe, Yoshiyuki Fukugawa, Natsuo Oya

**Affiliations:** Department of Radiation Oncology, Kumamoto University Hospital, 1-1-1, Honjo, Chuo-ku, Kumamoto‑shi, Kumamoto, 860-8556 Japan

**Keywords:** Adaptive radiation therapy, Glioblastoma, Clinical efficacy, Recurrence patterns

## Abstract

**Background:**

Glioblastoma (GBM) is an aggressive primary brain tumor with a high recurrence rate despite multimodal treatment approaches. Adaptive radiation therapy (ART) involves adjusting the treatment plan based on tumor and resection cavity changes during radiotherapy, potentially improving treatment precision while reducing radiation exposure to normal brain tissue. However, the clinical outcomes and recurrence patterns associated with ART remain unclear. We aimed to evaluate the efficacy of ART for GBM treatment, focusing on survival outcomes and recurrence patterns.

**Methods:**

We retrospectively analyzed a prospectively collected cohort of 59 patients with pathologically confirmed GBM who received postoperative three-dimensional conformal radiotherapy (3D-CRT)–based ART between April 2015 and November 2018. Mid-treatment magnetic resonance imaging was performed after delivery of 34–36 Gy. Based on these images, an offline single-time-point ART boost plan was generated to accommodate changes in tumor size and the resection cavity. Radiotherapy consisted of 40 Gy in 20 fractions to the initial target, followed by a 20 Gy boost in f10 fractions (total 60 Gy in 30 fractions over six weeks). Progression-free survival (PFS) and overall survival (OS) were estimated using the Kaplan–Meier method. Recurrence patterns were classified by the spatial relationship between recurrent tumor volume and the 95% isodose line.

**Results:**

During a median follow-up period of 19.2 (range, 2.1–81.6) months, 36 patients (61.0%) experienced tumor recurrence, and 32 (54.2%) died. The 1- and 2-year OS rates were 93.9% and 54.6%, respectively, with a median OS of 26.6 months. The 6- and 12-month PFS rates were 71.1% and 46.1%, respectively, with a median PFS of 10.5 months. Central recurrence was the most common pattern (29 patients, 78%), followed by distant (5 patients, 14%) and in-field recurrences (3 patients, 8%). Marginal recurrence was not observed. No cases of grade 2 or higher radiation necrosis were observed, and only two cases of grade 1 radiation necrosis were identified.

**Conclusions:**

ART for GBM is associated with favorable survival outcomes and low toxicity. ART does not increase the risk of marginal recurrence, and the incidence of radiation necrosis is low. Further studies are required to optimize ART protocols to maximize their clinical benefits.

## Background

 Glioblastoma (GBM) is the most common primary malignant brain tumor in adults and remains a significant treatment challenge, despite advances in imaging technologies and therapeutic approaches. The standard treatment for GBM consists of maximal surgical resection, followed by postoperative radiation therapy (RT) with temozolomide (TMZ). However, GBM is known for its therapy-resistant nature and high recurrence rate, with a median survival of approximately 15 months after diagnosis [1, 2]. Although multimodal treatments have modestly improved outcomes [3], the prognosis remains poor for most patients.

Radiotherapy for GBM is further complicated by dynamic changes in tumor volume and resection cavity during the treatment period [4–9]. For example, an increase in tumor volume during treatment can compromise target coverage if the original RT plan is followed. Conversely, if the resection cavity shrinks, the surrounding normal brain tissue may be exposed to radiation. These challenges highlight the importance of adapting radiotherapy to account for the structural changes during treatment.

Adaptive RT (ART) is a technique designed to address these challenges by adjusting the radiation treatment plan in response to changes in the tumor and surrounding tissues during treatment. This approach has the potential to improve the target dose coverage while reducing radiation exposure in normal brain tissues. In recent years, online ART using MR-linac platforms has also been introduced; the Phase II UNITED1 trial reported that margin reduction was feasible with a low marginal recurrence rate [10]. 

**Fig. 1:**
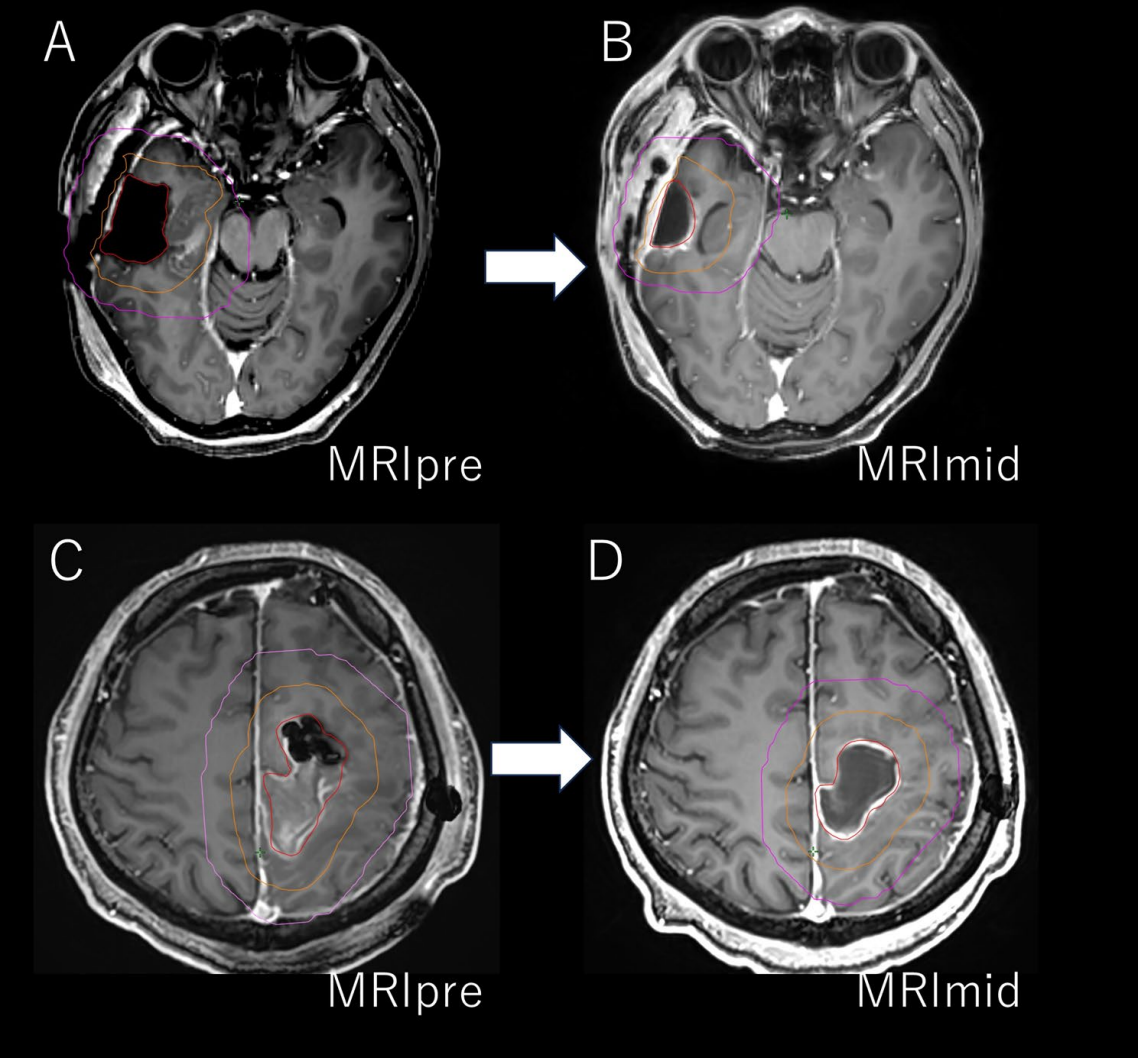
Representative examples of single-time-point offline adaptive boost planning. **A**–**B**: 42-year-old man, right temporal lobe glioblastoma. **A**: Baseline contrast-enhanced T1-weighted MRI (MRIpre) with resection cavity (red) and CTV (orange) contours and the corresponding 95% isodose line (magenta). **B**: Mid-treatment MRI (MRImid) showing resection-cavity (red) shrinkage and the adapted CTV (orange); the adapted 95% isodose line (magenta) encloses a 28% smaller volume than MRIpre. **C**–**D**: 53-year-old man, left parietal lobe glioblastoma. **C**: MRIpre with resection cavity (red) and CTV (orange) contours and the corresponding 95% isodose line (magenta). **D**: MRImid shows resection-cavity (red) shrinkage and the adapted CTV (orange); the adapted 95% isodose line (magenta) encloses a 40% smaller volume than MRIpre

In our previous prospective study [11], we demonstrated that ART could improve the dose distribution by incorporating mid-treatment magnetic resonance imaging (MRI) scans to adapt to treatment plans. These adjustments allowed for better coverage of the target volume and reduced radiation exposure to the healthy brain tissue (Fig. 1). Although promising, ART also raises certain challenges, such as the potential for radiation necrosis when the irradiation field is expanded or marginal recurrence when the field is excessively reduced.

To date, limited data are available on the clinical outcomes of ART in patients with GBM. This study aimed to evaluate the clinical outcomes and recurrence patterns associated with ART in patients with GBM. By focusing on these aspects, we hope to contribute to a better understanding of the role of ART in improving the treatment strategies for GBM.

## Materials and methods

### Patient selection

This retrospective analysis used a prospectively collected cohort of adult glioblastoma patients treated with ART at our institution. Although the ART protocol and delivery were predefined, treatment-outcome and recurrence-pattern evaluations were performed retrospectively, as these endpoints were not specified at study inception. The prospective cohort received IRB approval from Kumamoto University (IRB No. 1893).

Of 61 consecutive patients enrolled in the prospective ART study, two pediatric cases (< 18 years) were excluded due to distinct genetic profiles and prognoses, yielding 59 adult patients for analysis. All tumors were classified as WHO grade IV glioblastoma per the 2007 or 2016 WHO CNS tumor classifications. Patients underwent postoperative radiotherapy between April 2015 and November 2018 (Table 1).

**Table 1:** Patient characteristics (n=61)

Variable	
Sex	n(%)
Male	36(59)
Female	25(41)
Age (median, range)	64, 4–78
KPS	
< 70	18(30)
80–90	18(30)
90–100	25(41)
Extent of surgical resection	
GTR	31(51)
STR	23(38)
Bx	7(11)
Location of tumor	
Frontal	17(28)
Parietal	13(21)
Temporal	22(36)
Occipital	3(5)
Thalamus	2(3)
Cerebellum	2(3)
Multifocal	2(3)
O6-methylguanine-DNA methyl-transferase status	
Methylated	30(49)
Unmethylated	21(34)
Unknown	10(16)
IDH1-R132H immunohistochemistry	
Positive	3(5)
Negative	55(92)
Equivocal	1(2)
Unknown	1(2)
Months from surgery to radiation (median, range)	0.6 (0.3–4.1)

KPS, Karnofsky Performance Status; GTR, gross total resection; STR, subtotal resection.; Bx, biopsy only; IDH, Isocitrate dehydrogenase

We restricted the cohort to those receiving three-dimensional conformal radiotherapy (3D-CRT), excluding patients treated with intensity-modulated radiotherapy (IMRT) or volumetric-modulated arc therapy (VMAT). Although VMAT is now standard for postoperative GBM at our center, the original prospective study compared 3D-CRT preplans with 3D-CRT boost plans, and no VMAT/IMRT cases were enrolled in this analysis.

### Adaptive radiation therapy protocol

A summary of the offline, single-time-point ART used in this study is as follows. The clinical target volume initial (CTVinitial) was delineated as the high-signal area on T2-weighted imaging with a 1-cm margin, as observed on both preoperative and postoperative MRI scans. The planning target volume initial (PTVinitial) was defined by adding a 0.5-cm margin to the CTVinitial. A dose of 40 Gy, divided into 20 sessions, was initially administered to the PTVinitial using three or four beams, with one or two beams that were non-coplanar. All treatment plans were generated using the Eclipse treatment planning system (Varian Medical Systems, Palo Alto, CA, USA). The dose distribution was adjusted to ensure that 95% of the planning target volume (PTV) received at least 95% of the prescribed dose. Adjustments were made as necessary with respect to the dose constraints for organs at risk, such as the optic nerve and the eyes.

After delivering 34–36 Gy to the initial planning target volume (PTV_initial), a single offline mid-treatment contrast-enhanced MRI (MRI_mid) was obtained to generate the adaptive boost plan. On MRI_mid, the enhancement zone and resection cavity plus a 1 cm margin defined the clinical target volume boost (clinical target volume [CTV]_boost); adding a 0.5 cm margin around CTV_boost defined the planning target volume boost (PTV_boost). Hyperintense areas on T2-weighted/FLAIR images were included only if deemed residual tumor rather than edema. If the enhancing tumor decreased on MRI_mid, corresponding brain tissue from the pretreatment enhancement was retained in CTV_boost. Adaptive radiotherapy was applied to all patients regardless of change magnitude. PTV_boost then received a 20 Gy boost in 10 fractions. Thus, the initial PTV received 40 Gy in 20 fractions, followed by a 20 Gy boost in 10 fractions, totaling 60 Gy in 30 fractions over 6 weeks. All treatments were delivered on a Varian Clinac iX linear accelerator. Image guidance at each fraction used either the on-board imaging (OBI) system, orthogonal kV radiographs matched to cranial bone anatomy, or the ExacTrac stereoscopic kV imaging system (Brainlab AG, Munich, Germany). Patients were immobilized with a thermoplastic mask. Further plan details are provided in our previous report [11]. 

Concurrently, patients received oral TMZ according to the Stupp protocol [1]. 

### Posttreatment follow-up

After completing treatment, patients underwent contrast-enhanced MRI monthly and had regular follow-up visits with a neurosurgeon and radiation oncologist for six months. If no recurrence was detected during that period, MRI surveillance was then extended to every two months.

### Assessment of treatment response and recurrence patterns

In this retrospective study, we analyzed the treatment responses and recurrence patterns in GBM using the Response Assessment in Neuro-Oncology (RANO) criteria [12], specifically as follows: (1) Within the first 12 weeks post-RT, progression was defined as the appearance of new enhancing lesions in high-dose regions or beyond the 80% isodose line or by histopathologic evidence of a viable tumor through biopsy or reoperation. (2) Beyond 12 weeks post-RT, disease progression was defined as meeting any of the following criteria: (a) New enhancing lesions outside the radiation field while on steroid treatment; (b) An increase in lesion diameter of ≥ 25%; (c) Clinical deterioration; (d) Increase in T2/ non-enhancing lesions while on antiangiogenic therapy.

### Recurrence pattern classification

**Fig. 2:**
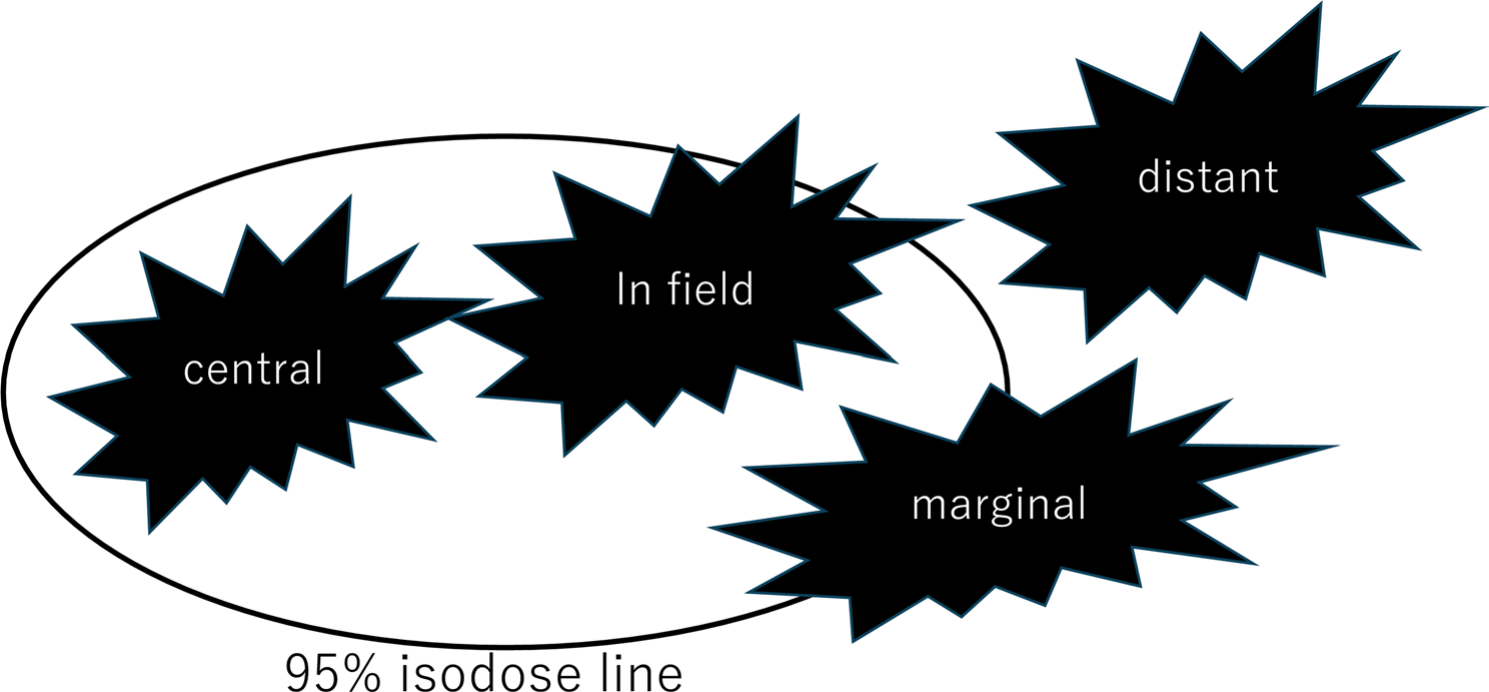
Recurrence patterns based on 95% isodose lines. Recurrence patterns were classified based on the overlap between the recurrent tumor volume on contrast-enhanced magnetic resonance imaging and the 95% isodose line (19 Gy) of the boost plan. Central recurrence: ≥95% overlap. In-field recurrence: 80–95% overlap. Marginal recurrence: 20–80% overlap. Distant recurrence: <20% overlap

If recurrence was observed, contrast-enhanced MRI was combined with boost treatment planning computed tomography using Eclipse. Recurrence patterns were classified into four categories based on the relationship between the recurrent tumor volume on contrast-enhanced MRI and the 95% isodose line (i.e., the 19 Gy line) of the boost treatment plan (Fig. 2). The classification was as follows: (1) Central recurrence: At least 95% of the recurrent tumor volume is located within the 95% isodose line. (2) In-field recurrence: Between 80% and 95% of recurrent tumors are located within the 95% isodose line. (3) Marginal recurrence: Between 20% and 80% of the recurrent tumor volume is within the 95% isodose line. (4) Distant recurrence: < 20% of the recurrent tumor volume is within the 95% isodose line.

### Survival analysis

Overall survival (OS) and progression-free survival (PFS) were assessed. OS was measured from initial surgery to death or last follow-up. PFS was defined as the interval from surgery to radiographic progression per RANO criteria or death, whichever occurred first. Both OS and PFS were estimated by the Kaplan–Meier method.

### Evaluation of central nervous system necrosis

Central nervous system necrosis was evaluated using the Common Terminology Criteria for Adverse Events, version 5.0.

### Ethical approval

This retrospective study was approved by the Ethics Committee of Kumamoto University Hospital (Approval Number: 3038).

## Results

**Fig. 3:**
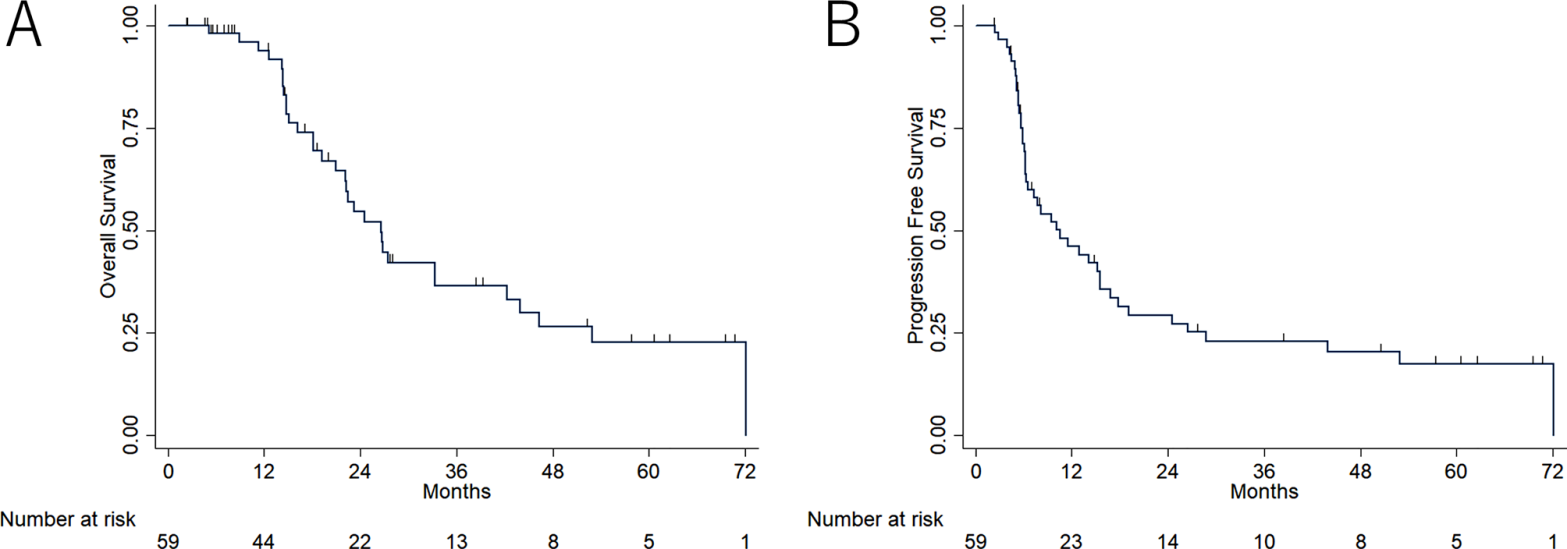
Kaplan–Meier survival curves for overall survival (OS) and progression-free survival (PFS). Kaplan–Meier survival curves of patients treated with adaptive radiation therapy for glioblastoma. (**A**) OS. (**B**) PFS

Data were censored on 5 April 2023. During the follow-up period, 32 (54.2%) deaths and 36 (61.0%) recurrences were confirmed (median follow-up period, 19.2 [range, 2.1–81.6] months). The 1-year OS rate was 93.9% (95% confidence interval [CI], 82.2–97.8), and the 2-year OS rate was 54.6% (95% CI, 38.7–68.0), with a median OS of 26.6 months. The 6- and 12-month PFS rates were 72.0% (95% CI, 58.6–82.0) and 46.1% (95% CI, 32.6–58.6), respectively, with a median PFS of 10.5 months. (Fig. 3).

Bevacizumab was administered to 31 patients after RT. Of these, three were started on bevacizumab at the start of RT for clinical research purposes, and the remaining patients received it at recurrence as part of salvage therapy. The duration of treatment varied according to clinical response. No cases of grade 2 or higher radiation necrosis were observed during the follow-up period. Radiation necrosis was suspected in two patients: one was diagnosed with grade 1 necrosis, and the other was suspected to have grade 1 necrosis.

Salvage surgery was performed on 11 patients, 2 of whom underwent re-irradiation following recurrence after surgery. In addition, one patient underwent re-irradiation without salvage surgery.

**Fig. 4:**
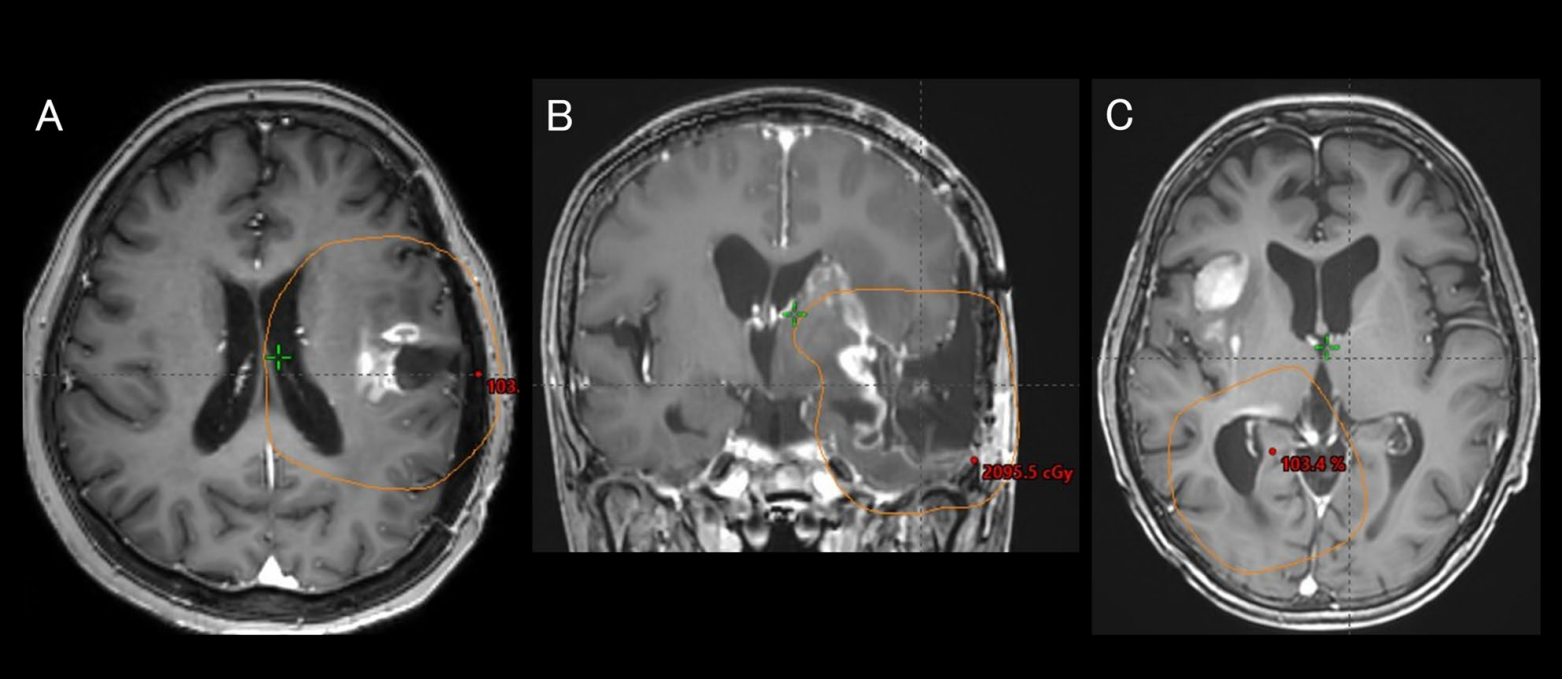
Recurrence patterns after adaptive radiation therapy. Magnetic resonance imaging images showing the recurrence patterns of glioblastoma. The orange line represents the 95% isodose line. (**A**) Central. (**B**) In-field. (**C**) Distant

The details of the recurrence patterns are listed in Table 2, and representative cases are illustrated in Fig. 4.

**Table 2:** Recurrence patterns

Recurrence type	n (%)	Median time to recurrence (Months)	Range (Months)
Central	29 (78)	6.2	1.9–28.7
Distant	5 (14)	14.8	4.3–15.5
In-field	3 (8)	3.9	3.0–10.1
Marginal	0	N/A	N/A

The most common initial recurrence pattern was central recurrence (29 patients), followed by distant (5 patients) and in-field (3 patients) recurrences. No marginal recurrences were observed. The median time from the initial surgery to the first recurrence was 6.2 (range, 1.9–28.7) months for central recurrence, 14.8 (range, 4.3–15.5) months for distant recurrence, and 3.9 (range, 3.0–10.1) months for in-field recurrence.

**Fig. 5:**
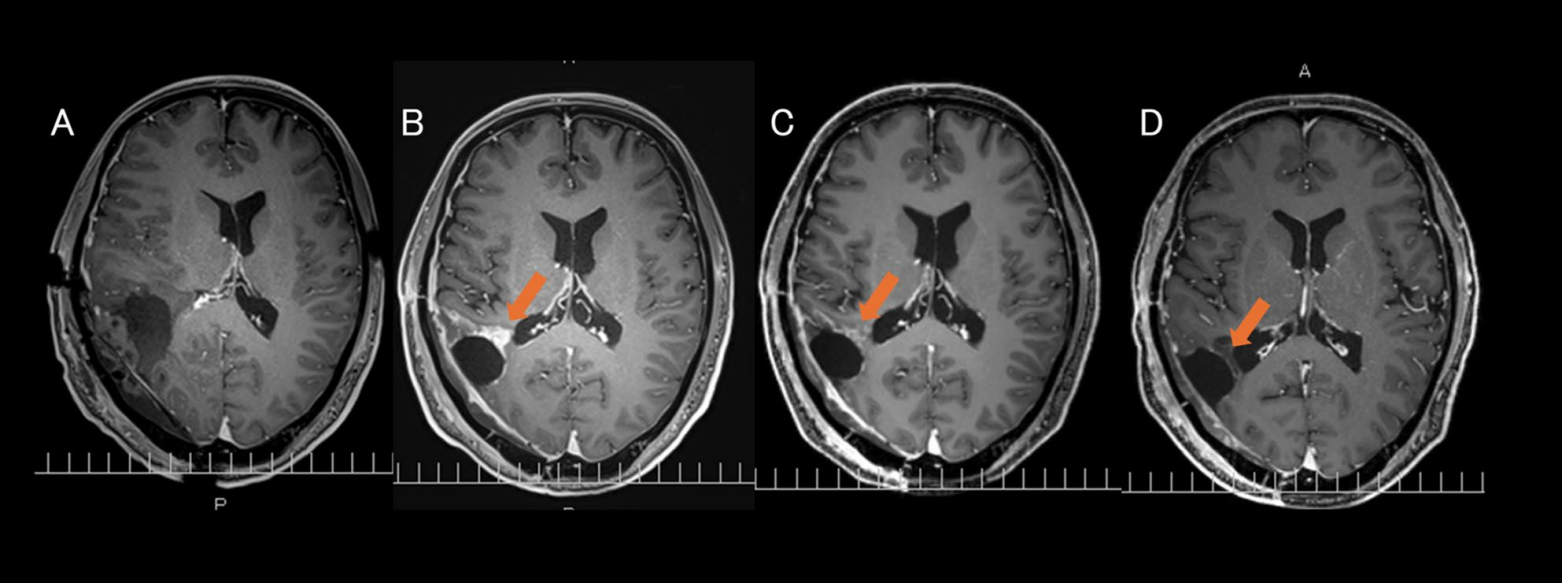
Suspected pseudoprogression during radiation therapy in a 45-year-old male patient. (**A**) Pretreatment. (**B**) Mid-treatment shows an apparent increase in enhancement. (**C**) End of radiation therapy, showing partial regression. (**D**) Two months posttreatment, showing further shrinkage, supporting pseudoprogression

During the RT period, 6 patients exhibited an expansion of the enhancement area on MRI, which subsequently shrank or disappeared. Among these, four patients did not show re-expansion of the enhancement area, suggesting the possibility of pseudoprogression during the RT period. Among these cases, an example of a 45-year-old male patient is shown in Fig. 5. Although the initially enlarged enhancement area decreased after RT, this patient later developed a central recurrence at a different site within the 95% isodose line.

## Discussion

The clinical outcomes of ART for glioblastoma in our cohort were comparable to, or exceeded, those reported in recent studies [1–3, 13]. GBM often exhibits residual tumor growth and cavity displacement between planning and treatment. In our prior prospective dosimetric study of 61 patients (including two pediatric cases later excluded from the present analysis) [11], ART improved target coverage while reducing radiation exposure to normal brain tissue. Specifically, gross tumor volume (GTV) was compared between n treatment initiation and mid-treatment MRI; mean ratios of mid-treatment to baseline GTV showed a significant decrease in the proportion of patients with GTR (0.84) and an increase in those without (1.30). As a result, in the GTR group, ART reduced the irradiated normal brain volume; for example, the median V20 (volume of normal brain receiving ≥ 20 Gy) decreased from 2.0 mL to 0.8 mL. Simultaneously, in the non-GTR group, ART improved target coverage, with median PTV V95 (the percentage of the PTV receiving at least 95% of the prescribed dose) increasing from 91.9% with non-ART plans to 98.2% with ART plans. Thus, the adaptive approach allows expansion of the treatment field when residual tumors grow or new lesions appear and contraction when the resection cavity shrinks. These favorable outcomes likely reflect reliable delivery of 60 Gy through timely replanning before the boost phase based on observed tumor changes. However, two study-specific factors may have influenced the favorable survival rates: First, exclusion of patients requiring IMRT or VMAT boosts, who had larger or more irregular tumors, resulted in a cohort well-suited to 3D-CRT; second, limited on-site follow-up for some patients may have affected survival estimates.

Our approach ensured that replanning was performed after 34–36 Gy, just before the final 20 Gy boost phase. However, the optimal timing for adaptive replanning in ART remains unclear and requires further investigation. Şenkesen et al. [9] performed replanning at approximately 42 Gy and demonstrated that this mid-treatment adjustment significantly improved tumor coverage while reducing doses to critical structures, such as the brainstem and optic chiasm. This emphasizes the importance of careful replanning to ensure that normal tissues are spared without compromising tumor control. In contrast, Stewart et al. [8] quantified tumor dynamics during concurrent chemoradiation and reported that significant tumor migration (> 5 mm) occurred in approximately 58% of the patients by the 10th fraction. These findings highlight the necessity for early intervention, as the tumor volume and position can change considerably within the first half of the treatment.

Bernchou et al. [7] demonstrated that large variations in GTV occurred early in the treatment course, often in the 10th fraction. Although they found that sufficient CTV margins prevented the need for replanning in their cohort, they noted that in cases of substantial tumor changes, failure to adapt the plan could lead to geographical misses and suboptimal tumor coverage. Given the significant variability in tumor dynamics observed during treatment, these studies collectively suggest that early replanning during the course of RT could be beneficial for certain patients. Further studies are required to refine the optimal timing and criteria for replanning to maximize therapeutic outcomes while minimizing risks to healthy tissues.

Although adaptive replanning offers significant benefits, a potential concern with ART is that reducing the irradiation field in response to tumor shrinkage may increase the risk of marginal recurrence. When the resection cavity or residual tumor contracts, shrinkage of the irradiation field may leave residual tumor cells at the margins, potentially leading to recurrence outside the treated area. Thus, balancing the benefits of reducing radiation exposure to healthy tissues with the risk of undertreating the tumor margins is crucial. In this study, central recurrence was the most frequent pattern, followed by distant and in-field recurrences; no marginal recurrence was observed. This finding is consistent with those of several previous studies that reported central recurrence as the predominant pattern in patients with GBM treated with RT. Ogura et al. [14] reported that central recurrence occurred in 66.7% of cases, with rare marginal recurrence. Similarly, McDonald et al. [15] demonstrated that PTV margins of ≤ 1 cm were not associated with an increased risk of marginal recurrence. This suggests that ART can effectively maintain tumor control while minimizing radiation exposure to normal tissues by avoiding unnecessarily large treatment margins. Gebhardt et al. [16] reported that of 95 documented recurrences, 81% had an in-field component, 6% a marginal component, and 28% a distant component. Notably, 66% of the patients experienced in-field-only recurrence, whereas marginal-only and distant-only recurrences accounted for 3% and 15%, respectively. In our study, despite using relatively small CTV margins with ART, the recurrence patterns were consistent with those reported previously. This outcome may be explained by the fact that even when mid-treatment MRI showed tumor shrinkage, the brain tissue where the tumor was originally present was still included in the GTV during replanning, ensuring comprehensive coverage of the potential residual tumor cells.

In addition to the risk of marginal recurrence, another concern with ART is the potential increase in neurotoxicity due to the expansion of the irradiation field in response to tumor growth or pseudoprogression [17]. If the MRI performed during replanning shows an increase in the enhancing region that is misinterpreted as true tumor progression, this could lead to unnecessary expansion of the irradiation field. Such expansions may increase the dose to the normal brain tissue, potentially elevating the risk of radiation necrosis. Pseudoprogression is commonly observed within the first few months after RT [17], but the possibility of its occurrence during the treatment period cannot be ruled out. This uncertainty complicates adaptive replanning, as the misinterpretation of pseudoprogression as tumor progression may lead to overtreatment and an increased risk of neurotoxicity. Despite these potential risks, the incidence of radiation-induced necrosis in our study was low. We did not observe any cases of symptomatic brain necrosis; only one case of grade 1 necrosis and one case of suspected grade 1 necrosis were identified in our study. This suggests that careful dose management and vigilant monitoring of dose–volume parameters in the normal brain can help mitigate the risk of significant neurotoxicity. However, because a substantial proportion of patients in this cohort received bevacizumab, which may have influenced the observed incidence of necrosis, further studies are warranted to clarify this association [18]. 

When considering the initial recurrence pattern and time to recurrence for each recurrence type, Ogura et al. reported that the median time to recurrence was longer for central/in-field recurrences than for outfield/distant recurrences [14]. In contrast, our study demonstrated a tendency for central/in-field recurrences to occur earlier than distant recurrences. This discrepancy may be related to differences in treatment approaches, particularly ART use. In ART, the irradiation field is redefined based on mid-treatment MRI, which sometimes reveals new lesions. As a result, recurrences that would have been classified as distant under a conventional radiotherapy approach may instead be counted as central or in-field in ART, provided they fall within the updated target volume. Consequently, distant recurrences in ART are more likely to represent truly late-emerging lesions, which may account for the observed delay in their appearance. On the other hand, Milano et al. [19] reported that distant recurrences tended to occur later than central or in-field recurrences, particularly among patients with longer survival durations. Differences in recurrence classification and evaluation criteria across studies may also contribute to these discrepancies. Further research is warranted to clarify the influence of ART on recurrence patterns.

In recent years, MRI-linac–delivered online ART has emerged as a promising extension of the offline approach. Detsky et al. [10] recently reported a weekly online-adaptive protocol for high-grade glioma in a single-arm, Phase II trial (NCT04726397). Despite using an exceptionally small CTV margin of 5 mm (with a 3 mm PTV margin), the marginal-recurrence rate was only 4%, demonstrating the feasibility of tighter margins when employing regular online adaptation. Ongoing multicenter trials will provide additional evidence: UNITED 2 is evaluating whether hypofractionated chemoradiotherapy can safely combine further margin reduction with dose escalation under weekly online adaptation [20]; UNITED 3 is assessing a two-phase adaptive schedule that incorporates detailed neurocognitive and quality-of-life endpoints [21]. In light of our findings, definitive results from these prospective trials of adaptive radiotherapy for glioblastoma are eagerly awaited to clarify optimal margins and the timing of replanning in both online and offline MRI-guided ART workflows.

During the RT period, six patients exhibited an initial expansion of the enhancing lesion on MRI, followed by shrinkage. Among these, four patients showed no subsequent re-expansion of the enhancing lesion, suggesting the possibility of pseudoprogression. Although it is also possible that these four patients initially progressed during the RT period and subsequently responded to RT, the absence of later re-expansion is consistent with the possibility of pseudoprogression. Pseudoprogression is typically reported to occur within three months after the completion of RT [17], but these cases indicate that it can also manifest during the treatment period. This observation provides new insights into pseudoprogression, suggesting that it may manifest during treatment in some cases. Expanding the irradiation field in response to pseudoprogression can lead to overtreatment or increased neurotoxicity. Therefore, careful interpretation of MRI changes is essential when considering adaptive replanning, and evaluating the dose to normal brain tissue is also important. In the future, as it becomes possible to accurately distinguish between pseudoprogression and true tumor progression, the quality of ART will improve.

This study has some limitations. First, this was a single institution retrospective study, meaning that the treatment strategies, eligibility criteria, and adaptive replanning protocols were specific to our institution. As this was a retrospective analysis, our study was subject to potential selection bias and data availability constraints. Additionally, the generalizability of our findings to other institutions remains uncertain, and further validation through multi-institutional prospective studies is required to confirm our findings. Furthermore, our study utilized relatively small CTV margins, which may have influenced the tumor control and recurrence patterns. Although our results suggest that ART with small margins does not increase the risk of marginal recurrence, further studies are required to evaluate whether similar outcomes can be achieved across different patient populations and treatment settings. Additionally, all treatment outcomes reported in this study were based on 3DCRT. Currently, IMRT and VMAT are widely used to treat postoperative GBM. Because these techniques achieve steeper dose gradients, tumor changes during treatment may have a greater impact on the dose distribution. Further evaluations of ART using IMRT and VMAT are needed.

Second, our toxicity assessment primarily focused on radiation necrosis; however, the potential impact of ART on cognitive function remains unclear. Although no cases of symptomatic radiation necrosis were observed, the retrospective nature of our study limited our ability to systematically evaluate the neurocognitive outcomes. The long-term effects of adaptive replanning, particularly regarding changes in irradiation volume and dose distribution, require further investigation. Future studies incorporating neurocognitive assessments are needed to better understand the potential risks of ART-related neurotoxicity.

## Conclusions

In this offline, single-time-point cohort, ART for GBM did not increase marginal-recurrence rates and maintained a low incidence of radiation necrosis. Moreover, ART yielded favorable clinical outcomes, including encouraging OS and progression-free survival rates. These results support ART as a safe and effective means of enhancing radiotherapy precision in GBM. Further research is needed to optimize ART protocols, particularly to establish optimal replanning intervals and assess long-term effects on tumor control and neurotoxicity.

## Data Availability

No datasets were generated or analysed during the current study.

## Abbreviations

3DCRT: Three-dimensional conformal radiotherapy
ART: Adaptive radiation therapy
CI: Confidence interval
CTVinitial: Clinical target volume initial
GBM: Glioblastoma
IMRT: Intensity-modulated radiotherapy
MRI: Magnetic resonance imaging
MRImid: Mid-treatment contrast-enhanced magnetic resonance imaging
OS: Overall survival
PFS: Progression-free survival
PTVinitial: Planning target volume initial
RANO: Response Assessment in Neuro-Oncology
RT: Radiation therapy
TMZ: Temozolomide
VMAT: Volumetric-modulated arc therapy
